# Actinomycin D-Activated RNase L Promotes H2A.X/H2B-Mediated DNA Damage and Apoptosis in Lung Cancer Cells

**DOI:** 10.3389/fonc.2019.01086

**Published:** 2019-10-25

**Authors:** Huijing Yin, Zhengyu Jiang, Shuoer Wang, Ping Zhang

**Affiliations:** ^1^Cancer Institute, Fudan University Shanghai Cancer Center, Shanghai, China; ^2^Department of Oncology, Shanghai Medical School, Fudan University, Shanghai, China; ^3^Department of Immunology, Tongji University School of Medicine, Shanghai, China; ^4^Faculty of Anesthesiology, Changhai Hospital, Second Military Medical University, Shanghai, China; ^5^Central Laboratory, The Fifth People's Hospital of Shanghai, Fudan University, Shanghai, China

**Keywords:** actinomycin D, RNase L, DNA damage, apoptosis, lung cancer

## Abstract

**Background:** Chemotherapy is an essential component for comprehensive cancer treatment, while drug resistance usually fails therapy. DNA repair mechanism of cancer cells restrains the efficacy of therapeutics targeting DNA damage. Investigating target-inducing irreversible cell death of cancer cells may be promising.

**Methods:** The present study used lung cancer cell lines, transplanted tumor model of lung cancers derived from patients with lung adenocarcinoma, and molecular experiments to investigate the effects and mechanism of Actinomycin D (Act D)-activated RNase L in lung canceers.

**Results:** We report that RNase L, when activated by Act D, induces Caspase-3/PARP activation. The latter further enables ROCK-1 to initiate subsequent membrane blebbing and, meanwhile, result in DNA cleavage and cell cycle arrest mediated by H2A.X/H2B-p21 axis, leading to irreversible DNA damage, and apoptosis of lung cancer cells. The present study highlighted the crucial role of RNase L in triggering apoptosis mechanism through the Caspase-3/ROCK-1/PARP/H2A.X+H2B/p21 axis during Act D treatment. Moreover, activation of RNase L suppressed the tumor formation and the induction of lung cancer stem cells.

**Conclusion:** This study unveiled the regulatory function and related mechanism of RNase L and implied the promising application of therapeutics targeting RNase L in lung cancer.

## Background

Chemotherapy has been recommended as an essential component for the comprehensive treatment for lung cancer (1). Several randomized control trials indicated the efficacy of adjuvant chemotherapy for the enhanced prognosis of non-small cell lung cancers (NSCLCs) (2). However, drug resistance usually leads to the failure of chemotherapy, especially in patients with NSCLCs. For example, cisplatin could induce DNA cleavage and cell death of cancer cells, whereas the DNA repair and related mechanism of cancer cells result in chemotherapy resistance and eventually lead to tumor recurrence. Therefore, developing new chemotherapeutics targeting DNA cleavage but also inhibiting repair mechanism of cancer cells may provide alternatives for the treatment of lung cancers.

Actinomycin D (Act D), an antibiotic and antineoplastic compound derived from *Streptomyces parvulus*, has been widely used for childhood tumors, women choriocarcinoma, and pancreatic cancer (3), while the effects of Act D in lung cancers are poorly investigated. The mechanism is that Act D interacts with guanine and cytosine base pairs in DNA through the phenoxazone ring and forms hydrogen binds that blocks the chain elongation (4, 5). Act D also externally binds to DNA, terminal GC base pairs, or double- and single-strain DNA with no GC binding site (4, 6). Several studies also reported the mechanism of its anti-tumor effects through inhibiting RNA polymerases and decreases transcription, resulting in the death of tumor cells (5, 7). However, comprehensive and detailed mechanism about how Act D induces cell death of cancer remains largely unknown. On the other hand, clinical trials using Act D synergized with other anti-cancer agents have achieved promising results in controlling cancer progression, whereas the dose-limiting toxic effects of Act D, including hepatic toxicity (8), bone marrow depression (9), low platelet, immune suppression, and gastrointestinal issues, hinder its efficacy and application in cancer management (10). Our preliminary research found that a low dose of Act D presented varied efficacy to different tumor cells, implicating that a specific molecule may influence drug sensitivity of Act D. We hypothesize that low-dose Act D may induce the activation of specific target or signaling, which may promote DNA damage, amplify the apoptosis-induction effects of Act D, and, at the same time, reduce the toxic effects. Therefore, investigating the mechanism of anti-tumor effects of Act D and developing targeted therapy for specific intermediated molecule should be a promising strategy for cancer treatment.

Previous researches discovered RNase L as an essential component in interferon (IFN)-mediated antiviral signaling, which mainly targets the RNA substrate to initiate cellular defense against the virus (11). Also, RNase L could activate macrophage-related immune responses to eliminate viral infections (12). Upon activation, RNase L acts as an endoribonuclease that catalyzes single-strain RNA (ssRNA) or rRNA (13), inhibiting virus replication and inducing cell apoptosis. Except for the function in anti-viral immunity, RNase L could also regulate proliferation (14), influencing apoptosis (15) and autophagy (16), indicating a broader function of RNase L apart from the anti-viral effects in the interferon system. However, whether RNase L could suppress the progression of lung cancer, or whether it could be regulated by Act D to initiate anti-tumor effects, is poorly investigated.

In the present study, we first report that Act D induces, in an RNase L-dependent manner, DNA cleavage, and cell cycle arrest at G2/M. Mechanistically, we found that Act D activates RNase L and induced signaling of the Caspase-3/ROCK-1/PARP/H2A.X+H2B/p21 axis. More importantly, activation of RNase L suppressed the tumor formation, and the induction of lung cancer stem cells. Thus, the present study reported that RNase L, a key regulator in immune responses, induces DNA cleavage and apoptosis of cancer cells, suggesting the application of Act D as a novel chemotherapeutic for cancer treatment.

## Methods

### Cell Culture and Reagents

The human lung adenocarcinoma cell line NCI-H460 was kindly provided by Stem Cell Bank, Chinese Academy of Sciences. Cells were cultured in DMEM (Hyclone, USA) supplemented with 10% fetal bovine serum (FBS) (Gibco, USA) and 1% penicillin–streptomycin (Hyclone, USA). Caspase-3 inhibitor (z-DEVD-fmk), PARP inhibitor (3-AB), and ROCK-1 inhibitor (Y-27632) were purchased from Calbiochem, USA. Act D (BioVision, USA) was used for dose dependence and time course.

### Human Lung Cancer Tissue Dissociation and Cell Line Establishment

Human lung cancer tissues were obtained from the Fudan University Shanghai Cancer Center, with the approval of the hospital ethical committee. Lung tissues were collected during surgery, and samples were preserved in pre-cooled 1,640 culture medium supplemented with 2% penicillin–streptomycin solution (Sigma, USA). The following procedures were conducted in ice: connective tissue and vascular were removed from lung cancer tissue and washed with PBS; then, tissue was dissected off and minced into small pieces in a conical tube containing 3 ml of PBS, 60 μl of collagenase/dispase (10269638001, Roche), and 7.5 μl of 1% DNase I (D4527, Sigma) followed by rotating incubation for 45 min at 37°C. The cells were then filtered sequentially through 100- and 40-μm strainers and centrifuged at 800 rpm for 5 min at 4°C. The cell pellet was resuspended in 1 ml of red blood cell lysis buffer (R7757, Sigma) for 1 min and washed in PBS.

Cell line establishment: cells were then resuspended with culture medium supplemented with 20% FBS and 2% penicillin–streptomycin solution and planked in a culture plate. After 4 h of adherence, the culture medium was changed to a normal medium and cultured ever since.

### siRNA Interference

siRNA of RNase L (sc-45965), Histone H2A.X (sc-62464), Histone H2B (sc-105522), and control siRNA (sc-37007) were purchased from Santa Cruz and diluted to 10 μM with TE buffer. Lipofectamine® RNAiMAX (Invitrogen, USA) was used for siRNA transfection. Cells were seeded at a six-well culture plate overnight (1 × 10^6^/well for WB, 1 × 10^4^/well for ICF), and the culture medium was then changed to Opti-MEM. The transfection system was prepared as follows: 9 μl of Lipofectamine® RNAiMAX was diluted in 150 μl of Opti-MEM and then mixed with siRNA-Opti-MEM. After 5 min of mixing, the transfection system was then added to wells.

### Construction of Plasmids for RNase L Silencing, Overexpression, and Establishment of Stable Cell Lines

To silence RNase L expression, we synthesized DNA oligos for the transcription of specific shRNAs designed to target *RNase L*-specific mRNAs and were inserted into plko.1/puromycin vectors. Also, a scrambled shRNA was used as the negative control. Primers of RNase L shRNA were as follows: rnase l-sh-1: Forward: 5′-CCGGCAGACTCTGGAAGCGTGTTTGCTCGAGCAAACACGCTTCCAGAGTCTGTTTTTG-3′; Reverse: 5′-AATTCAAAAACAGACTCTGGAAGCGTGTTTGCTCGAGCAAACACGCTTCCAGAGTCTG-3′. rnase l-sh-2: Forward: 5′-CCGGGACAATCACTTGCTGATTAAACTCGAGTTTAATCAGCAAGTGATTGTCTTTTTG-3′; Reverse: 5′-AATTCAAAAAGACAATCACTTGCTGATTAAACTCGAGTTTAATCAGCAAGTGATTGTCT-3′. rnase l-sh-3: Forward: 5′-CCGGCTGAAGGATCTCCACAGAATACTCGAGTATTCTGTGGAGATCCTTCAGTTTTTG-3′; Reverse: 5′-AATTCAAAAACTGAAGGATCTCCACAGAATACTCGAGTATTCTGTGGAGATCCTTCAGT-3′. cDNAs for *RNase L* were amplified from NCI-H460 by reverse transcription (RT)-PCR using Nest PCR primers and inserted into the PCDH-puromycin vectors. Primers of RNase L cDNA were as follows: Outside (forward): 5′-ACG CGT GAC GTC GTA AGG CCT CCA GC-3′; (reverse): 5′-ACG CGT GAC GTC GTA AGG CCT CCA GC-3′. Inside (forward): 5′-ACG CGT GAC GTC GTA AGG CCT CCA GC-3′; (reverse): 5′-ACG CGT GAC GTC GTA AGG CCT CCA GC-3′. Silencing of *RNase L* expression by shRNA in the cell line obtained from human lung cancer tissue (Sample #2). The overexpression of *RNase L* was performed in the cell line obtained from human lung cancer tissue (Sample #6). All transfection was conducted through lentivirus-mediated delivery. The cells were selected with puromycin at 2 μg/ml for 5 days. All control cell lines were generated by infection with viruses containing the empty vector or a scrambled shRNA vector following the same protocol.

### RNase L Dimerization Assay

Cells were washed with pre-cooling PBS and then loading buffer [20 mM Tris–HCl (pH 8.0), 137 mM NaCl, 10% glycerol, 1% NP-40, and 2 mM EDTA] was added to lysate cells. Then, Cocktails buffer (a mixture of protease inhibitor and phospholipase inhibitor) (Roche, USA) was added to the cell lysate and was incubated on ice for 20 min and centrifuged at 4°C at 12,000 rpm for 15 min. The supernatant was collected and mixed with 5 mg/ml DMS (dimethyl suberimidate) (Fluka, USA) and incubated at room temperature for 30 min. Then, samples were mixed with isometric SDS loading buffer and subjected to SDS-PAGE for Western blot.

### Detection of RNase L Activity

This measurement referred to the method reported by Rusch et al. (17). Cells were lysed with TRIzol, and then with 200 μl of chloroform, centrifuged at 12,000 rpm at 4°C for 10 min and the supernatant was collected. Then 500 μl of isopropanol was added, mixed, cooled on ice for 20 min, and centrifuged at 4°C at 12,000 rpm for 10 min. The supernatant was discarded and 1 ml of 70% ethanol was added for washing. Then, 20 μl of TMC buffer [10 mM Tris–HCl (pH 7.5), 5 mM MgCl_2_, and 100 nM CsCl] was added to reconstitute RNA and immediately subjected to RNA electrophoresis. Cleavage products and 18S and 28S rRNAs were observed under UV exposure. RNase L activity was measured quantitatively according to the cleavage products of rRNA.

### Western Blot

The protein was separated on a sodium dodecyl sulfate–polyacrylamide gel electrophoresis, transferred to PVDF membranes (Millipore, USA), and blocked with 5% non-fat dry milk in TBST. After washing three times with TBST, the following primary antibodies dissolved in antibody buffer (Keygentec, China) were used: anti-RNase L (Abcam, USA), anti-phosphor-H2A.X (Serl39) (Millipore, USA), anti-phosphor-H2B (Serl4) (Millipore, USA), anti-H2A.X (Abcam, USA), anti-H2B (Abcam, USA), anti-ROCK-1 (Abcam, USA), anti-Caspase-3 (Cell Signaling Technology, USA), anti-PARP (Cell Signaling Technology, USA), anti-p21 (Cell Signaling Technology, USA), and anti-β-actin (Cell Signaling Technology, USA). After the secondary antibody incubation, the membrane was washed three times with TBST and exposed with ECL (Millipore, USA). The corresponding semi-quantitative analysis was performed by measuring the optical density using ImageJ software.

### Flow Cytometry

Mouse lung tissue was digested to single-cell suspension, and 1 × 10^6^ cells were prepared for CD166 staining (PE-CD166, 560903, BD Pharming) or control antibody (IgG kappa Isotype control). Cells were stained at 4°C for 30 min, avoiding light. Then, cells were washed with PBS, centrifuged, resuspended with 100 μl of PBS, and subjected to flow cytometry analysis.

### Immunohistochemistry (IHC)

The sections were deparaffinized, hydrated, and antigen-retrieved using retrieval solution. The sections were then quenched with 0.3% hydrogen peroxide in methanol for 30 min to block endogenous peroxidase activity and washed with TBS (pH 7.2). Subsequently, the sections were blocked with 5% normal goat serum for 20 min and then incubated with primary antibodies against Ki-67 (#9449, CST) overnight at 4°C in a humid incubator. After washing with TBS, the sections were incubated with biotinylated goat anti-mouse (115-065-003,1:1,000, Jackson ImmunoResearch Laboratories) antibodies for 2 h in a humidified incubator. The signal was detected using the avidin-biotin-peroxidase complex (PK-6100, Vector Laboratories, Burlingame, CA) in combination with the DAB substrate (SK-4100, Vector Laboratories, Burlingame, CA), followed by a wash with TBS-T (pH 7.4). Finally, the sections were rinsed in distilled water, counterstained with hematoxylin (H-3401, Vector Laboratories, Burlingame, CA), and mounted on microscope slides.

### Terminal-Deoxynucleotidyl Transferase Mediated Nick End Labeling (TUNEL) Analysis

For ICF TUNEL staining: cell slides were fixed with 1% paraformaldehyde at room temperature for 10 min and then pre-cooled with ethanol/acetic acid (2:1) at −20°C for 5 min. After PBS washing, slides were incubated with terminal deoxynucleotidyl transferase (TdT) buffer (Millipore, USA) at 37°C for 1 h and washed three times with a stop solution for 15 s and PBS. Then, slides were incubated with rhodamine-conjugated anti-digoxigenin antibody (Millipore, USA) at 37°C for 30 min, washed with PBS, stained with DAPI for 2 min, and washed with PBS. Slides were subjected to microscopy, and the images were merged by Image J.

For IHC TUNEL staining: the sections were deparaffinized, hydrated, and incubated with proteinase K (10 μg/ml) at 37°C for 30 min. Then, plates were washed with PBS and reacted with TUNEL analysis solution (*In Situ* Cell Death Detection Kit, Roche, USA) in a humidified incubator at 37°C for 1 h. After PBS washing, Converter-POD was added and incubated for another 30 min. The plates were then incubated with DAB substrate at 25°C for 20 min, avoiding light. After PBS washing and hematoxylin staining, the plates were then dehydrated, Made transparent, and mounted for microscopy.

### Cell Cycle Analysis

Cells at the logarithmic phase were trypsinized and planked at 2 × 10^6^ overnight. Then, the culture medium was changed to RPMI1640 containing 0.2% FBS and cultured for 48 h. Now, cells were synchronized at the G0/G1 phase. Later culture medium was changed to RPMI1640 containing 10% FBS and cultured for another 20 h. After 20 h culturing, cells were pre-treated with si-RNase L or si-control for 48 h and then treated without or with 0.1 μg/ml Act D for the indicated times. Cells were then calculated, resuspended, and eventually fixed with pre-cooling 70% ethanol at −20°C overnight. Then, cells were washed with PBS, and 500 μl of PI staining buffer was added and incubated at room temperature for 30 min, avoiding light. After incubation, cells were subjected to flow cytometry [FACS Calibur (BD USA)], and data were analyzed by WinMDI ver 2.9. Cell cycle percentage was examined with Cylchred 1.0.2.

### Proliferation Assay

Cells were pre-treated with si-RNase L or si-control for 48 h and then treated with or without 0.1 μg/ml Act D for 72 h. Then, cells were incubated with BrdU at 10 μmol/L for 24 h in a cell incubator. Cells were then harvested by cell scraper and centrifuged at 1,200 rpm for 5 min. After washing three times, cells were fixed with 4% PFA for 30 min and washed with PBS. Then, 1% Triton X-100 was used for permeabilization for 20 min. After PBS washing, cells were incubated with DNase at 300 μg/ml for 1 h and then centrifuged at 1,200 rpm for 5 min. Then BrdU antibodies were added, incubated with cells for 30 min at room temperature, and subjected to flow cytometry.

### Annexin V/PI Staining

Cells were pre-treated with si-RNase L or si-control for 48 h and then treated with or without 0.1 μg/ml Act D for the indicated times. Cells were harvested and resuspended with 100 μl of Annexin V binding buffer (Molecular Probes, USA) and incubated at room temperature for 5 min. Then 5 μl of Annexin V-FITC and 5 μl of PI were added, mixed and incubated at room temperature for 15 min, avoiding light. For flow cytometry analysis, cells were subjected to FACS Calibur (BD, USA) and analyzed with WinMDI ver 2.9 software. For microscopy, 20 μl of cell suspension was added between the cover glass and glass slide for observation, and images were analyzed with ImageJ.

### Mouse Tumor Growth Assays and Intratumoral Injection

For the xenograft tumor growth, 8-week-old NOD-SCID mice were maintained in a pathogen-free environment. Briefly, the cells were harvested by trypsinization, washed twice with PBS, and then resuspended in PBS. Six mice were used per cell line, and each mouse received bilateral subcutaneous injections of 1 × 10^6^ cells in 150 μl of PBS for each injection. After 2 weeks, Act D was injected intratumorally. The tumor is divided into three points, and each point is injected sequentially. The total volume of Act D is 150 μl, and the needle only slowly entered 1/3 of the tumor.

The dose of ActD is 3 mg/kg, which is injected once every 3 days for 2 weeks. Tumor growth was monitored every 3 days, and the tumor volume was calculated by the following formula: tumor volume (in mm^3^) = *a* × *b*^2^ × 0.52. All mouse experiments were approved by the Institutional Animal Care and Use Committee of Fudan University and performed following institutional guidelines and protocols.

### Tail Vein Injection

A total of 1 × 10^5^ cells in 100 μl of DMEM was injected to 8-week-old NOD-SCID mice through tail vein injection. Two weeks later, Act D was injected intraperitoneally. The dose of ActD was 3 mg/kg, which was injected once every 3 days for 2 weeks. After waiting for another 4 weeks, the mice were euthanized with CO_2_, the thoracic cavity was opened, and the lungs were completely removed by clamping the trachea. Under the same treatment conditions, a part of the lungs was used for fixation, embedding, sectioning, and staining. After a portion of the tissue was digested, a single-cell suspension was prepared for flow analysis.

### Statistical Analysis

Data were expressed as mean ± SEM. Student's *t* test was conducted for comparisons between two groups, and one-way ANOVA was performed for comparisons among several groups. A *P* value <0.05 was considered to be statistically significant.

## Results

### Act D Induces the Activation of RNase L and Activates Caspase-3/PARP-Mediated Apoptosis

**Figure 1:**
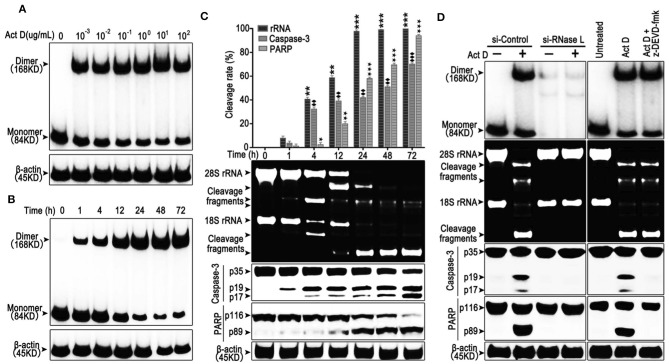
Actinomycin D (Act D) promotes RNase L dimerization and RNase L-dependent apoptosis in lung cancer cells. **(A,B)** Western blot of dose dependence (10^−3^-10^2^ μg/ml) (0.8–8 × 10^4^ nM) **(A)** and time course **(B)** of Act D-induced RNase L dimerization. **(C)** Cleavage activity of RNase L and Western blot of activated Caspase-3 and PARP after Act D (0.1 μg/ml) (80 nM) treatment in the indicated time. **(D)** Cleavage activity of RNase L, Western blot of RNase L dimerization and activated Caspase-3 and PARP after RNase L-knock down (si-RNase L) or Caspase-3 inhibitor (z-DEVD-fmk) with the treatment of Act D (0.1 μg/ml) (80 nM) for 24 h. Data were collected from triplicate samples in three independent experiments. ★*P* < 0.05; **, ♦♦, ★★*P* < 0.01;***, ♦♦♦, ★★★*P* < 0.001.

RNase L functions as a homodimer (18). To investigate whether Act D could activate RNase L, we applied Act D at different dosages to evaluate the dimerization of RNase L and found that the dimerization of RNase L achieved the highest level when Act D was used at 10 μg/ml (Figure 1A). Further research proved that the dosage range from 0.1 to 10 μg/ml could elevate the dimerization of RNase L to a plateau. As we focused on the effects of Act D in low dosage, we applied 0.1 μg/ml in further experiments. At this dosage, we discovered the time curve of RNase L activation and found that the dimerization within 72 h increased with time and reached a plateau after 48 h (Figure 1B).

The activation of RNase L would lead to the catalyzation of rRNA and catalyzed rRNA further, as a damage signal, induce apoptosis (13, 19). Therefore, we investigated the effects of Act D-induced RNase L to the cleavage of rRNA and apoptosis-related marker, Caspase-3, and PARP. As the results showed, compared to 1 h, cleavage of rRNA, Caspase-3, and PARP is increased in a time-dependent manner (Figure 1C). The cleavage of rRNA occurred within 1 h after Act D treatment and peaked at 24 h. Moreover, the cleavage of Caspase-3 and PARP occurred 1 h later but was still increasing after 24 h (Figure 1C). Therefore, we further asked the activation order of these molecules. As the results showed, the RNase L-siRNA led to the impaired dimerization of RNase L and, meanwhile, inhibited the cleavage of rRNA, Caspase-3 and PARP (Figure 1D), indicating that the activation of RNase L is essential for the cleavage of Caspase-3 and PARP. Moreover, inhibition of Caspase-3 led to the suppression of PARP cleavage, indicating Caspase-3 functioning as the up-stream of PARP (Figure 1D). Thus, these results showed that Act D could activate RNase L and further promote Caspase-3/PARP-mediated apoptosis.

### Act D-Induced RNase L/Caspase-3/PARP Activation Promotes DNA Cleavage, H2A.X Activation, and H2B-Mediated DNA Condensation

**Figure 2:**
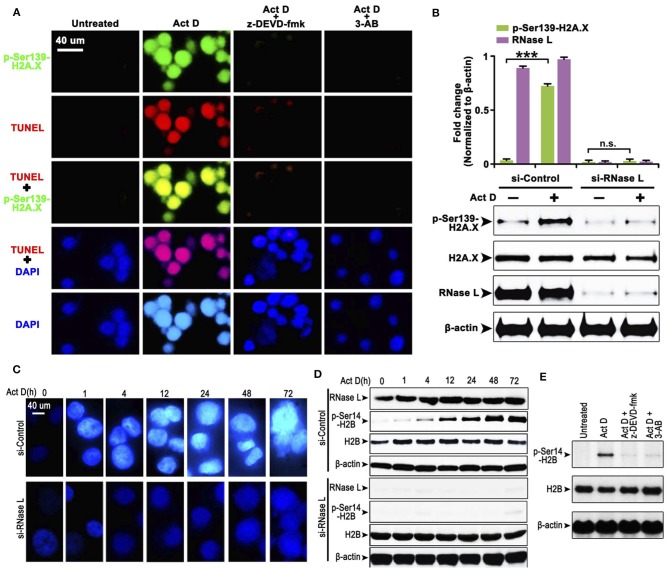
RNase L induces DNA cleavage and condensation with H2A.X-H2B activation through Caspase-3/PARP activation. **(A)** Immunocytofluorescence (ICF) of p-Ser139-H2A.X, TUNEL, and DAPI in NCI-H460 treated with Act D and Caspase-3 inhibitor (z-DEVD-fmk) or PARP inhibitor (3-AB). **(B)** Western blot of p-Ser139-H2A.X and H2A.X after RNase L interference. **(C)** ICF of DAPI in NCI-H460 treated with Act D in the indicated time. **(D)** Western blot of RNase L, p-Ser14-H2B, and H2B treated with Act D in the indicated time after RNase L interference. **(E)** Western blot of p-Ser14-H2B and H2B treated with Act D and Caspase-3 inhibitor (z-DEVD-fmk) or PARP inhibitor (3-AB). Data were collected from triplicate samples in three independent experiments. ***P < 0.001.

The cleavage of PARP indicates that the DNA lacks protection, which could be easily damaged and activates the phosphorylation of H2A.X at Ser139 to initiate the DNA repair process (20). We asked whether this process is accompanied by Act D-induced alteration of RNase L, Caspase-3, and PARP. We found that the Act D treatment promoted DNA cleavage, activated the phosphorylation of H2A.X-Ser139, and was suppressed if an inhibitor of Caspase-3 (z-DEVD-fmk) or PARP (3-AB) was applied (Figure 2A), indicating that Act D-induced Caspase-3/PARP activation promoted the DNA cleavage and further activated H2A.X. As RNase L is essential for the activation of Caspase-3/PARP, we also investigated whether the phosphorylation for H2A.X is also dependent on RNase L. The results showed that p-H2A.X-Ser139 was inhibited after RNase L interference while expression of H2A.X was not altered, indicating that the phosphorylation of H2A.X was dependent on RNase L (Figure 2B).

The result from Figure 2A showed that DNA (presented by DAPI staining) condensation was accompanied by DNA breakage. We asked whether DNA condensation is also an RNase L-dependent process and specific mechanism related. To answer this question, we firstly showed that DNA condensation was suppressed after RNase L interference (Figure 2C), which suggested an essential role of RNase L in Act D-induced DNA condensation. Moreover, as previous researches proposed that phosphor-H2B-Ser14-mediated DNA condensation is accompanied by phosphor-H2A.X after DNA cleavage (21), we investigated the alteration of H2B after Act D treatment. We found the phosphorylation of H2B increased following the level of DNA condensation, and RNase L interference inhibited the phosphor-H2B elevation (Figure 2D), indicating that RNase L promoted DNA condensation through H2B activation. We further proved that this process was also dependent on Caspase-3/PARP activation as the inhibitor of these two molecules inhibited the phosphorylation of H2B (Figure 2E). Thus, these results indicated that Act D activated RNase L/Caspase-3/PARP cascade and promoted the H2B-mediated DNA condensation.

### RNase L Activation Promotes Cell Cycle Arrest at G2/M, Inhibits Proliferation, and Induces Apoptosis

**Figure 3:**
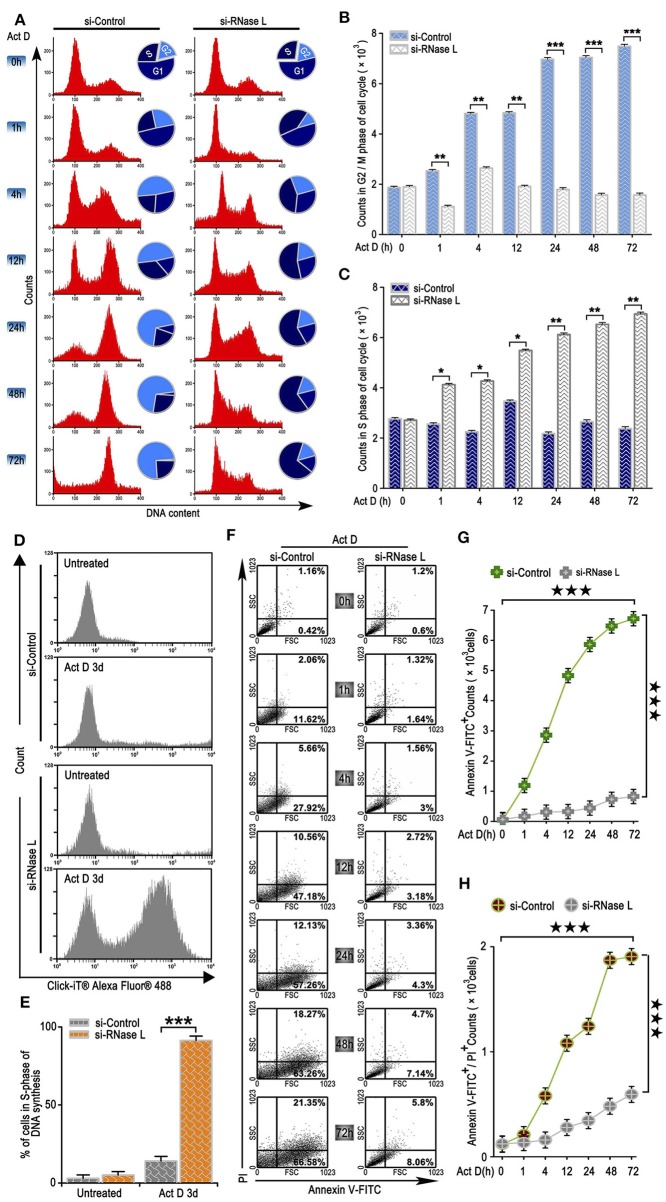
RNase L activation leads to cell cycle arrest at G2/M, suppresses DNA replication, and induces apoptosis in lung cancer cells. **(A–C)** Flow cytometry analysis of cell cycle **(A)** and analysis of G2/M **(B)** and S **(C)** phase cell counts treated with Act D after RNase L interference. **(D,E)** Flow cytometry of Alexa Fluor 488 in NCI-H460 treated with Act D for 3 days after RNase L interference. **(F–H)** Flow cytometry of Annexin V and PI **(F)**, and analysis of Annexin V^+^ **(G)**, and Annexin V^+^PI^+^ **(H)** in NCI-H460 treated with Act D for the indicated time after RNase L interference. Data were collected from triplicate samples in three independent experiments. *P < 0.05; **P < 0.01; ***, ★★★*P* < 0.001.

We further analyzed the effects of the irreversible DNA cleavage to cell cycle and apoptosis. We found that cell cycle was arrested at G2/M phase when RNase L was activated, while, in the RNase L interference group, cell cycle accumulated at S phase (Figures 3A–C). We then asked whether the accumulation of cells at S phase was due to the Act D-induced acceleration of DNA replication in RNase L deficiency. Through BrdU experiments, we found that DNA replication was accelerated after RNase L interference (Figures 3D,E). These results indicated that Act D-induced RNase L activation promotes DNA cleavage, suppressed DNA replication, and arrested cell cycle at G2/M phase. We further investigated whether RNase L activation induces cell apoptosis after the cell cycle arrest. As the results showed by Annexin V/PI staining in flow cytometry, Annexin V^+^ cells, as well as Annexin V^+^ PI^+^, increased after Act D treatment in a time-dependent manner in the control group while maintaining a relatively low level in the si-RNase L group (Figures 3F–H). These results indicated that the RNase L activation induces cell apoptosis.

### RNase L Induces Apoptosis Through Caspase-3/ROCK-1/PARP/H2A.X-H2B/p21 Cascade

**Figure 4:**
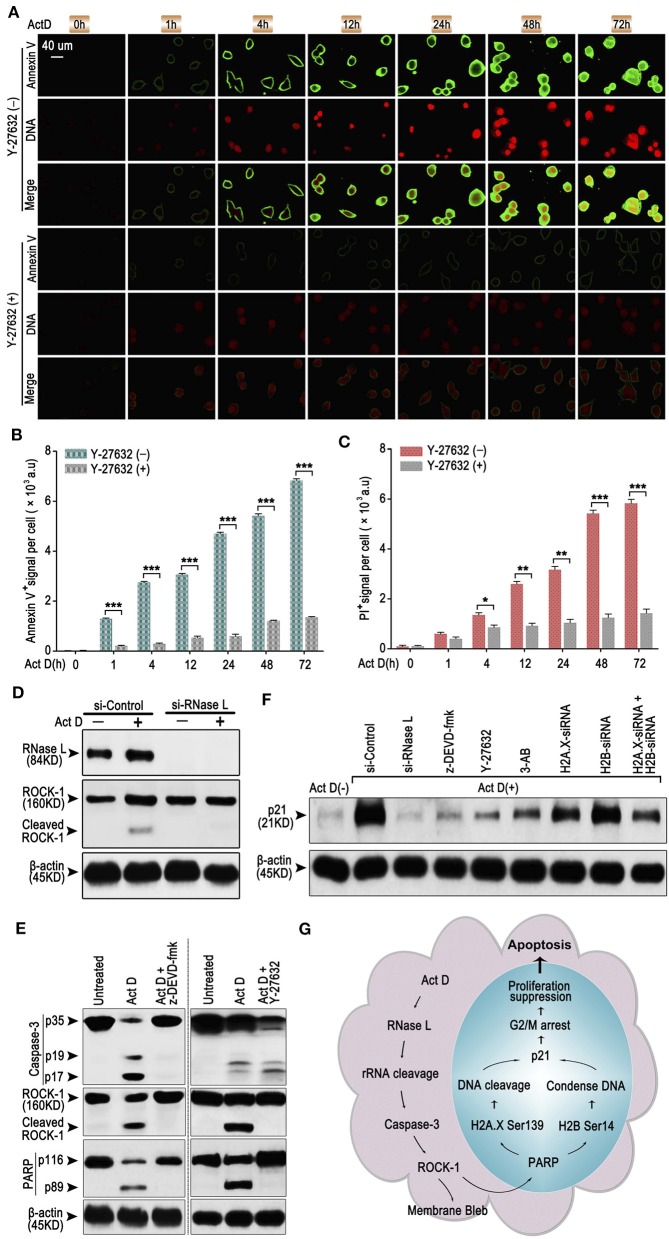
RNase L induces apoptosis through Caspase-3/ROCK-1/PARP/H2A.X-H2B/p21 cascade. **(A–C)** ICF of Annexin V and PI (DNA) **(A)** and analysis of Annexin V^+^ **(B)** or PI^+^ **(C)** in NCI-H460 in the presence of Act D with or without ROCK-1 inhibitor (Y-27632) for the indicated time. **(D)** Western blot of RNase L and ROCK-1 in NCI-H460 treated with Act D after RNase L interference. **(E)** Western blot of Caspase-3, ROCK-1, and PARP in the presence of Act D with or without Caspase-3 inhibitor (z-DEVD-fmk) or ROCK-1 inhibitor (Y-27632). **(F)** Western blot of p21 in NCI-H460 treated with Act D and different intervention [siRNA interference for RNase L, H2A.X, H2B or H2A.X, and H2B combined, Caspase-3 inhibitor (z-DEVD-fmk), ROCK-1 inhibitor (Y-27632), and PARP inhibitor (3-AB)]. **(G)** Graphical summary for Act D-activated RNase L-mediated apoptosis through Caspase 3-ROCK 1-PARP-H2A.X/H2B-p21 cascade. Data were collected from triplicate samples in three independent experiments. *P < 0.05; **P < 0.01; ***P < 0.001.

As an early sign of apoptosis, exposure of phosphatidylserine on the cell surface, which could be marked by Annexin V, is reported to be associated with the activation of ROCK-1 (22). We asked whether Annexin V^+^ is regulated by a particular regulatory molecule. We analyzed the Annexin V and PI staining through ICF and showed that the inhibitor of ROCK-1 significantly impaired the Act D-induced phosphatidylserine exposure and DNA cleavage (Figures 4A–C), suggesting that the activation of ROCK-1 is a critical process during Act D-induced apoptosis.

We further asked whether ROCK-1 is regulated by RNase L and how RNase L functions during the process of apoptosis. Activation of ROCK-1 could be marked by the cleaved part from the C terminal (130 kDa). Therefore, we first analyzed the ROCK-1 activation after RNase L interference and proved that the activation of ROCK-1 was dependent on RNase L activation (Figure 4D). Then, we investigated whether ROCK-1 acts as an essential part of RNase L/Caspase-3/PARP cascade. By applying inhibitor of Caspase-3 and ROCK-1, we found that an inhibitor of Caspase-3 impaired the activation of ROCK-1 and PARP while the inhibitor of ROCK-1 only reduced the activation of PARP (Figure 4E), indicating ROCK-1 act as an intermediate between Caspase-3 and PARP.

RNase L activation cleaved PARP and induced DNA damage and caused cell cycle arrest at G2/M phase. Cell cycle arrest is accompanies by elevated expression of a specific protein related to cell cycle regulation, such as p21 (23). Therefore, we investigated whether RNase L affected the expression of p21 and the possible role of p21 in RNase L regulatory mechanism. We found that Act D enhanced the expression of p21 while si-RNase L, si-H2A.X, si-H2B, si-H2A.X+si-H2B, and inhibitor of Caspase-3, ROCK-1, and PARP all resulted in the attenuated expression of p21 (Figure 4F), indicating p21 as downstream of Caspase-3/ROCK-1/PARP/H2A.X-H2B cascade.

Collectively, these results showed the regulatory mechanism that Act D-induced RNase L activation induces rRNA cleavage and Caspase-3/ROCK-1/PARP activation, which further activates H2A.X, H2B, and p21, leading to DNA cleavage, cell cycle arrest, inhibited proliferation, and promoted apoptosis (Figure 4G).

### RNase L Activated by Act D Suppresses Tumor Growth

**Figure 5:**
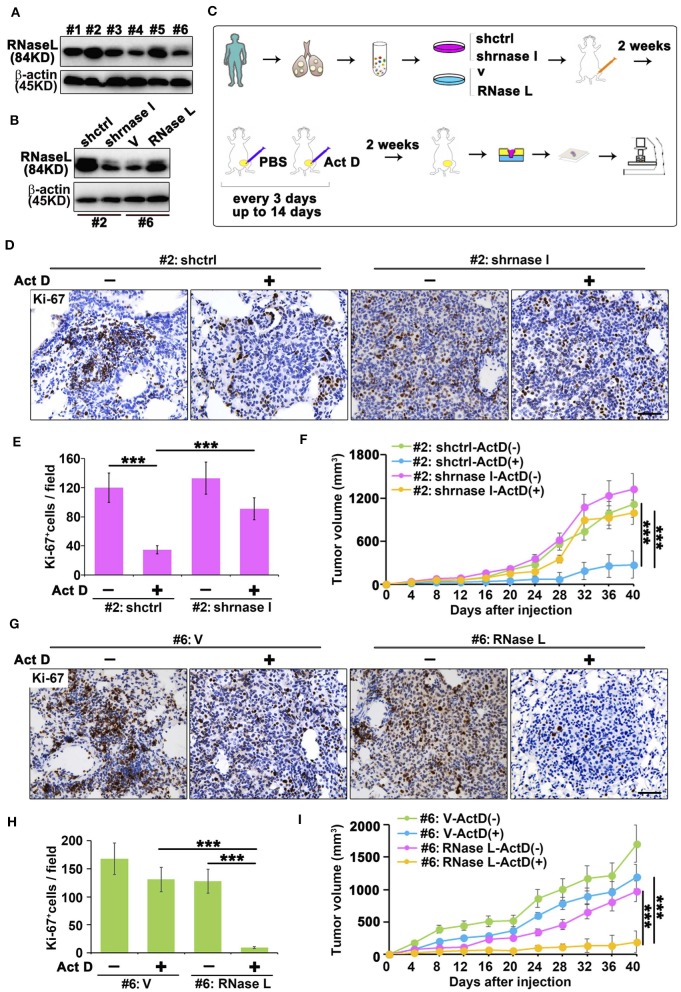
RNase L activated by Act D suppress tumor growth *in vivo*. **(A)** RNase L expression of six samples from cell lines derived from patients with lung adenocarcinoma (#1 to #6 represent the order of sample collections). **(B)** Expression of RNase L after overexpression or knockdown in lung cancer cell lines derived from patient's lung adenocarcinoma tissue. **(C)** Flowchart of animal experiments evaluating the activation of RNase L to tumor growth. **(D,E,G,H)** Ki-67 staining **(D,G)** and quantitative analysis **(E,H)** of transplantation tumor of human lung adenocarcinoma after knockdown **(D,E)** or overexpression **(G,H)** of RNase L. **(F,I)** Tumor volume measurement of transplantation tumor of human lung adenocarcinoma after knockdown **(F)** or overexpression **(I)** of RNase L. Data were collected from triplicate samples in three independent experiments. ***P < 0.001.

We collected the sample from patients with lung adenocarcinoma and found that RNase L presented different expression features among samples (Figure 5A). To investigate the effects of RNase L on tumor growth, we knocked out the RNase L in samples with elevated expression and overexpressed RNase L in samples with decreased expression (Figure 5B). Through the *in vivo* experiments proposed in Figure 5C, we found that, in samples with high expression of RNase L, Act D could activate RNase L and suppress the tumor growth whereas the knockdown of RNase L relieved the suppression to tumor growth (Figure 5F). Similarly, Act D treatment did not suppress the tumor growth in samples with low expression of RNase L while the suppression of Act D occurred after RNase L was overexpressed (Figure 5I). These results indicated Act D suppresses tumor growth through RNase L. Moreover, we used Ki-67 to evaluate the proliferation of tumor cells and found that Act D could suppress the proliferation in RNase L-high expressed or RNase L-overexpressed samples (Figures 5D,E,G,H), while Act D showed limited effects in RNase L-low expressed or RNase L-knockdown samples (Figures 5D,E,G,H). Thus, these results indicated that RNase L activated by Act D suppresses tumor growth through inhibiting the proliferation of tumor cells.

### RNase L Deficiency Promotes the *in situ* Enrichment of Cancer Stem Cells With Drug-Resistant and Anti-apoptotic Potentials in Lung Tumor

**Figure 6:**
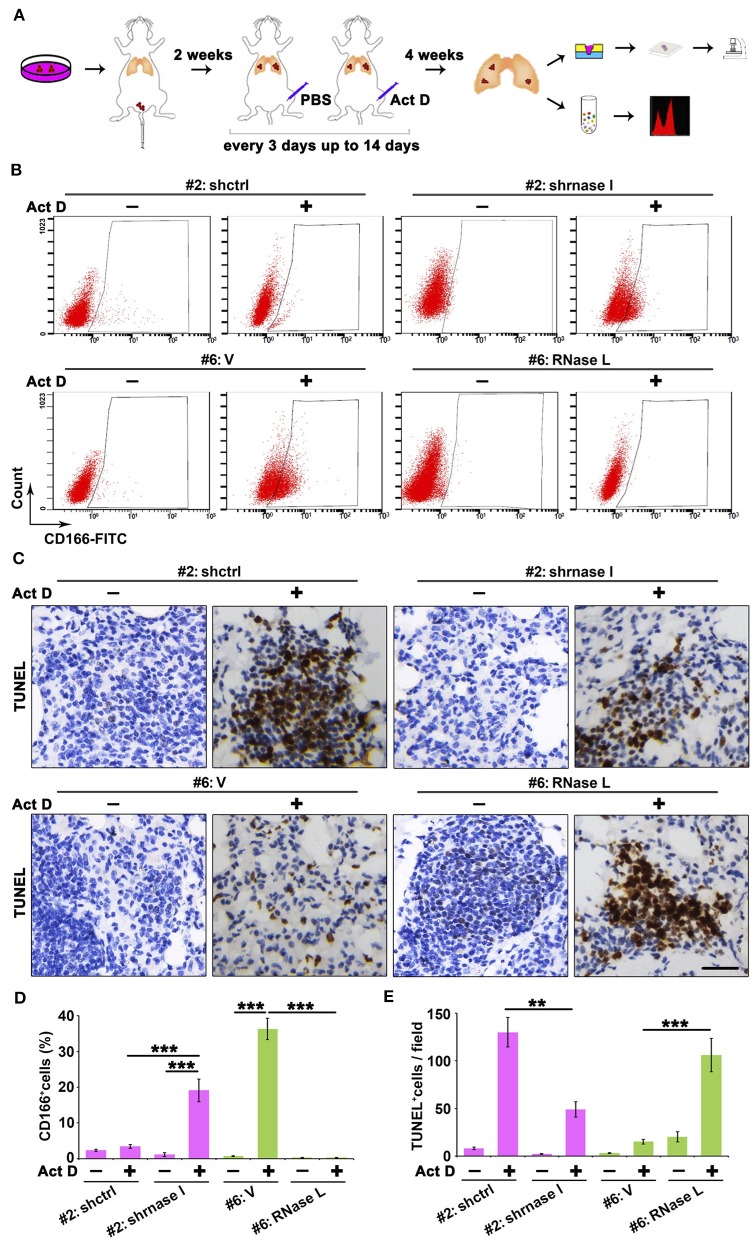
RNase L deficiency promotes the *in situ* enrichment of cancer stem cells with drug-resistant and anti-apoptotic potentials in lung tumors. **(A)** Flowchart of animal procedures. **(B,D)** Flow cytometry analysis of CD166^+^ cells **(B)** and quantitative analysis **(D)** after RNase L deficiency or overexpression. **(C,E)** IHC **(C)** and quantitative analysis **(E)** of apoptosis in the tumor of human-derived lung cancer cells after RNase L deficiency or overexpression. Data were collected from triplicate samples in three independent experiments. **P < 0.01; ***P < 0.001.

Results from Figures 5D–I indicated that the RNase L-deficiency promotes cell proliferation when treated with Act D, and this result is in accordance with Figures 3D,E, indicating the drug resistance of cancer cells. Drug resistance of cancer cells is usually associated with the function of cancer stem cells, and CD166 was proposed as a specific marker for cancer stem cells of lung adenocarcinoma (24). Through tail vein injection, we transferred the cells to the lung of NOD-SCID mice to evaluate the tumor-initiating capacity in the lung of human lung cancer-derived cells. We further used Act D to investigate the effects of RNase L activation to the tumor initiation capacity of these cells (Figure 6A).

We found that RNase L deficiency promoted the enrichment of CD166^+^ cells *in situ* tumor of lung (Figures 6B,D) with anti-apoptotic potentials (Figures 6C,E). On the other hand, the activation of RNase L suppressed the initiation of lung cancer stem cells and promoted apoptosis (Figures 6B–E).

Collectively, results from Figures 5, 6 proved that the activation of RNase L could suppress the tumor growth through inhibiting the proliferation of cancer cells, initiation of cancer stem cells, and promoting apoptosis of cancer cells.

## Discussion

Comprehensive treatment for cancer includes surgery, chemotherapy, radiation, target therapy, and immunotherapy. Except for the operation and radiation that uses exogenous elimination of cancer cells, chemo, targeted, and immunotherapy are all based on the understanding of malignant phenotypes and intrinsic regulation of cancer cells that induces cancer elimination through self-initiated or intercellular-induced cell death. Therefore, investigating molecules with a specific mechanism that possess the potential in suppressing proliferation and inducing apoptosis in cancer cells may provide indications for further development of integrated cancer therapy. In the present study, we found that RNase L, a crucial component in anti-viral immunity, functions as an initiator of apoptosis and related mechanism. RNase L activates the Caspase-3/ ROCK-1/PARP/ H2A.X+H2B/p21 axis and induces cell cycle arrest and apoptosis in lung cancer cells.

Recent advances in cancer therapy highlighted the molecule regulating immune responses functions as a tumor suppressor, such as PD-L1 activating T cells and promoting apoptosis of cancer cells (25, 26). Similarly, though RNase L mainly functions in anti-viral immunity (27, 28), recent literature also discovered its essential role in anti-proliferation and promoting apoptosis (29). However, the mechanism of RNase L reported only proposed several related signaling or cellular processes while the specific mechanism is poorly investigated (30, 31). In the present study, we highlighted the crucial role of RNase L as an initiator and dependent molecule in promoting apoptosis in lung cancer cells. Ribosome damage could act as a damage signal to activate apoptosis (32, 33), and ribosome cleavage induced by RNase L activation promotes Caspase-dependent apoptosis. These results indicated that the treatment targeting ribosome damage to initiate apoptosis in cancer cells might be an applicable method for cancer therapy. More importantly, our study showed the activation of RNase L not only induced rRNA cleavage but also caused DNA cleavage and inhibition of DNA replication, leading to irreversible damage and terminating transcription and translation. Therefore, RNase L activation could promote a “collapse” of the nucleic acid system and consequential cell death, indicating the valuable utility of targeting RNase L in lung cancer therapy.

Chemotherapy against NSCLCs frequently failed due to the drug resistance, especially for medicine mediating DNA cleavage in cancer cells (34, 35). This failure is usually due to the activated DNA repair system that restrains the efficacy of DNA-targeting chemotherapeutics. Similarly, in investigating the effects of Act D in RNase L deficiency, we also found that Act D promoted DNA synthesis and apoptosis inhibition, which suggested the activated DNA repair system after Act D treatment. However, this phenomenon was reversed after RNase L activation. The irreversible DNA cleavage induced by RNase L promotes apoptosis, indicating that chemotherapy combined with RNase L-targeting therapeutics may achieve promising results in lung cancer therapy.

An essential feature of apoptosis is the blebbing of phosphatidylserine, while little is known about the regulatory mechanism of this process. A recent study indicates that the activation of ROCK-1 could lead to the blebbing of phosphatidylserine and promote apoptosis of myocardial cell and pulmonary arterial smooth muscle cell (36, 37). However, this regulation is poorly investigated in cancer cells. In this study, we reported ROCK-1 activation, mediated by RNase L, resulting in the blebbing of phosphatidylserine in lung cancer cells and indicated this process as an essential mechanism in apoptosis. The specific function of ROCK-1 in cancer cells remains controversial. Recent studies proposed that it acts as an oncogene that regulates cellular movement and cancer metastasis (38, 39). However, there is also a study that discovered its role in promoting apoptosis of cancer cells (40). The present finding of RNase L activating ROCK-1 and inducing subsequent apoptosis provides further evidence of the specific function of ROCK-1 in cancer cells, thus indicating the crucial role of ROCK-1 activation in mediating apoptosis of lung cancer cells.

## Conclusion

The present study unveiled that Act D, an antibiotic and antineoplastic compound, could induce DNA cleavage cell cycle arrest and apoptosis in an RNase L-dependent manner. Mechanistically, Act D initiates RNase L and leads to subsequent activation of the Caspase-3/ROCK-1/ PARP /H2A.X+H2B/p21 axis. More importantly, the activation of RNase L could suppress tumor initiation and the enrichment of cancer stem cells, highlighting the RNase L as a critical initiator of apoptosis in lung cancer cells and suggesting promising therapeutics targeting RNase L as a novel treatment for lung cancer.

## Data Availability Statement

All data and materials are provided in the manuscript and figures.

## Ethics Statement

The studies involving human participants were reviewed and approved by Ethical Committee of Fudan University Shanghai Cancer Center. The patients/participants provided their written informed consent to participate in this study. The animal study was reviewed and approved by Ethical Committee of Fudan University Shanghai Cancer Center.

## Consent for Publication

All authors read the manuscript and consent for the publication.

## Author Contributions

HY and ZJ: cellular and molecular experiments and analysis and ICF and IHC analysis. HY and PZ: FACS assay. HY and SW: animal experiments. ZJ and HY: manuscript preparation. HY: project conception, scientific oversight, and input.

### Conflict of Interest

The authors declare that the research was conducted in the absence of any commercial or financial relationships that could be construed as a potential conflict of interest.

## Abbreviations

NSCLCs: Non-small cell lung cancers
Act D: Actinomycin D
ssRNA: Single-strain RNA
FBS: Fetal bovine serum
IHC: Immunohistochemistry
ICF: Immunocytofluorescence
TUNEL: Terminal-deoxynucleotidyl Transferase Mediated Nick End Labeling.
